# Urban-rural disparities in living arrangement and older adults care service supply in China: a cross-sectional study

**DOI:** 10.3389/fpubh.2026.1732990

**Published:** 2026-04-20

**Authors:** Penglei Xiao, Xiaoxiao Wang, Dan Xu, Yanfei Gao

**Affiliations:** 1 Department of Medical Record, Henan Provincial People's Hospital, Zhengzhou, Henan, China; 2 Research Center of Clinical Epidemiology, Peking University Third Hospital, Haidian District, Beijing, China

**Keywords:** older adults care services, healthy aging, living arrangement, supply and demand, urban-rural disparities

## Abstract

**Background:**

China's rapidly aging population was placing growing pressure on both families and the government to ensure adequate older adults care. Consequently, delivering comprehensive and high-quality services for the aged had become a pressing social issue.

**Objective:**

This study aimed to examine the living arrangement and older adults care service supply among older adults in China, identify urban-rural disparities, and explore potential directions for the future development of older adults care services.

**Methods:**

Data were obtained from the 2018 cross-sectional dataset of CLHLS, 15,087 older adults were included for analysis. Differences in the distribution of categorical characteristics among older adults with different living arrangement and hukou types were first examined. Logistic regression models were employed to identify factors associated with living arrangements. Differences in the distribution of availability of social security and of older adults care services between older adults with different hukou types were also examined by χ^2^ test and logistic regression.

**Results:**

Most of the participants (80.41%, *n* = 12,131) lived with house member(s), and 46.45% (*n* = 6,970) indicated that their main source of financial support came form their child(ren). Compared with rural older adults, urban older adults were more likely to be in an institution (*OR* = 4.782, 95% *CI* = 3.795–6.025, *P* < 0.001), less likely to be alone (*OR* = 0.694, 95% *CI* = 0.603–0.799, *P* < 0.001). As age increased, they were less likely to be alone or in in an institution (*OR* = 0.950, 95% *CI* = 0.945–0.954, *P* < 0.001; *OR* = 0.984, 95% *CI* = 0.974–0.994, *P* = 0.001). Male were more likely to be alone or in an institution than female (*OR* = 1.190, 95% *CI* = 1.072–1.322, *P* = 0.001; *OR* = 1.332, 95% *CI* = 1.095–1.622, *P* = 0.004). There were urban-rural disparities in the accessibility of older adults care services among older adults (*P* < 0.001).

**Conclusions:**

It was found that the primary living arrangement for older adults in China was co-residence with family members. And significant urban-rural differences existed, primarily reflected in economic status, insurance systems, and the availability of older adults care services. In particular, hukou type exerted a statistically significant effect on older adults care service supply.

## Introduction

### Definition of aging society

According to United Nations standards, a country or region is considered to have entered an aging society when the proportion of people aged 60 and above exceeds 10%, or when the proportion of people aged 65 and above exceeds 7% (1). This standard was established by the 1982 Vienna World Conference on Aging (2).

### Global aging status and driving factors

According to World Bank data, the proportion of the global population aged 65 and above has continued to rise from 1990 to 2023, and is positively correlated with national income levels (3). The driving factors for this trend varied depending on the economic level of the country (4). For low-middle income countries, aging is mainly due to young people's deaths, especially childhood and birth mortality, and higher mortality from infectious diseases, leading to a gradual shift in population structure toward aging. For high income countries, it is mainly attributed to a significant increase in life expectancy (such as Japan, Germany, and other countries where life expectancy has exceeded 80 years).

China is experiencing the world's largest and fastest aging process. At the end of 2024, the proportion of the population aged 60 and above in China reached 22.0% (approximately 310 million people), of which the proportion of the population aged 65 and above was 15.6%, far exceeding the international aging standard (5). It is worth noting that among the 31 provinces in China, 12 provinces have even entered the stage of “deep aging” (with a population aged 65 and above accounting for ≥ 14%), such as Liaoning and Shanghai.

### Multidimensional challenges of aging society

The aforementioned demographic changes has exerted increasing challenges to both individuals and government systems. Currently, the major challenges faced by the government and society could be summarized as follows:

(I) Labor challenge. The proportion of the eligible labor force has declined, resulting in a reduction in labor supply and a potential slowdown in economic productivity.
(II) Growing economic pressure. The substantial decline in the labor capacity and income of older adults heightens their dependence on public financial support. Consequently, the pension system faces a significant funding gap and become a heavy fiscal burden on the government, which in turn constrains other public expenditures.
(III) Imbalance in care service development. There is a severe mismatch between the supply and demand of older adults care services. Current care provision struggles to strike a balance among affordability (price), accessibility (availability), and quality (service level).
(IV) Transformation of inter-generational family relationships. The growth of the older adults population has directly increased care-giving responsibilities within families—particularly for spouses and adult children—thus reshaping inter-generational relationships and weakening the traditional family-based eldercare function.

### Realistic challenges faced by the older adults

Aforementioned demographic shift has also placed increasing pressure on individuals to meet older adults care service needs. The theory of needs is often applied to understand the actual needs of the older adults and discover ways to solve problems they faced (6). Based on previous researches, it is known that in the field of older adults care services, the needs of the older adults mainly focused on service security needs, environmental quality needs, and social participation needs (7).

Older adults people generally face the dilemma of being unable to afford premium care services while also struggling to find cost-effective alternatives in daily care services. Regarding environmental quality requirements, the primary challenge lies in the decline of health management and physical activity capabilities. For instance, falls have become the main cause of accidental injuries among the older adults, with about 30% of people aged 65 and over falling at least once a year (8). The resulting “fear of falling” further restricted their mobility, thereby heightening their need for safety and convenience in daily living environments. In terms of social participation, the older adults commonly encountered social exclusion and limited engagement in community activities. Empirical studies had shown that active socialization can delay epigenetic aging (9). Moreover, interpersonal communication—particularly family interaction—had been found to reduce cognitive decline among older adults (10). However, restricted mobility led to a shrinking social network, exacerbated social isolation, and thereby undermined the overall wellbeing of the older adults population.

In addition, the older adults were facing a mental health crisis. Researches shown that the incidence rate of depression in the older adults living alone was 40% higher than that in the general population (11), and social isolation would accelerate cognitive decline (12). However, the problems of older adults depression and anxiety caused by empty nest, loneliness, and social disconnection were seriously underestimated, and effective social support networks was a lacked.

These issues mentioned above had been deeply concerned and researched by scholars, various studies and explorations had been conducted on them. However, it cannot be denied that there are still other serious challenges in the lives of the older adults. For example, the demand for modern health care services of the older adults challenges the unbalanced and insufficient development of traditional care services, and the urban-rural differences in the distribution of older adults care services.

### Urban-rural disparities in older adults care services

Previous research had revealed a pronounced urban-rural dichotomy within China's older adults care service system (13). From a demographic perspective, the degree of population aging in rural areas exceeded that observed in urban regions. The proportion of older adults people aged 60 and 65 years and above in rural areas was 23.81 and 17.72%, respectively, which was 7.99 and 6.61% higher than those in urban areas (5), indicating that the rural older adults were facing a more severe aging situation and heavier care-giving burden than those in urban areas.

Economically, the financial pressure associated with older adults care was substantially greater for rural residents. According to recent data from the National Bureau of Statistics, the average replacement rate of enterprise employee pensions declined to 39.2% in 2024 (14). Rural older adults primarily relied on familial supports; however, the household savings rate had been continuously decreasing in recent years (15). Data from the Ministry of Human Resources and Social Security showed that the average monthly pension for rural residents in 2024 was approximately 200 CNY—far from sufficient to meet basic living expenses—whereas the average monthly pension for urban retirees exceeded 3,000 CNY. Based on this estimation, the actual pension gap between rural and urban older adults populations was thus approximately 15-fold (16).

In addition to the economic disparities, substantial differences existed in the allocation and availability of older adults care resources between urban and rural areas (17). Studies had demonstrated that the long-term care needs of older adults in rural areas were both more pressing and extensive. Furthermore, rural older adults individuals exhibited stronger anticipated needs for community-based support, reflecting their increasing dependence on external care resources (18).

Meanwhile, existing evidence further suggested that disparities between urban and rural older adults care institutions were widening both across regions and within provinces, thereby exacerbating spatial and structural inequalities in service provision (19). Generally speaking, older adults care facilities in urban areas tended to be relatively comprehensive, while it remained inadequate in rural areas. Hence, community-based older adults care had become the dominant form of support in urban areas, though the system remained fragmented, with uneven service distribution and a persistent shortage of qualified nursing professionals. By contrast, rural areas faced underdeveloped infrastructure, limited access to formal care institutions, and a gradual erosion of traditional family-based care-giving, driven by large-scale urban migration and progressive population aging.

The continuous acceleration of China's aging population had led to an increasing burden on individuals and the government in terms of older adults care security. Consequently, providing comprehensive and high-quality old-age-services had become a critical societal concern. Compounded by the legacy of the single-child policy, the rise in empty nesters within this aging society meant many older adults face significant challenges in securing adequate social, emotional, and economic support (11). Therefore, providing comprehensive and high-quality older adults services had become a key social issue, and imbalanced and insufficient development currently represented the core problem within China's pension and eldercare service systems (20).

Despite the extensive research on population aging in China, empirical evidence simultaneously comparing the supply and demand of older adults care services across urban and rural settings using nationally representative data remains limited. In light of the foregoing challenges, this study undertook a systematic comparative analysis of urban-rural disparities in China's older adults care services across multiple dimensions, including economic security, service accessibility, and institutional support mechanisms. A cross-sectional design allows for a comprehensive snapshot of current living arrangement and service availability among older adults, enabling urban-rural comparisons at the population level. This study aimed to: (1) describe living arrangement and older adults care service availability among older adults in China; (2) compare urban-rural differences in service supply and demand; and (3) identify gaps to inform community-based older adults care policies.

### Methodology

#### Study design and participants

This is a nationwide population-based cross-sectional study using data from the Chinese Longitudinal Healthy Longevity Survey (CLHLS), which was organized by the Development Research Center of Health and Aging (the National Development Research Institute) of Peking University. The survey covered 23 provinces, autonomous regions, and municipalities (Beijing, Tianjin, Hebei, Shanxi, Liaoning, Jilin, Heilongjiang, Shanghai, Jiangsu, Zhejiang, Anhui, Fujian, Jiangxi, Shandong, Henan, Hubei, Hunan, Guangdong, Guangxi, Hainan, Chongqing, Sichuan, Shaanxi. In this study, these provinces were categorized into four economic zones according to their economic status and geographical location: Eastern Region: Beijing, Tianjin, Hebei, Shanghai, Jiangsu, Zhejiang, Fujian, Shandong, Guangdong, Hainan. Central Region: Shanxi, Anhui, Jiangxi, Henan, Hubei, Hunan. Western Region: Guangxi, Chongqing, Sichuan, Shaanxi. Northeast Region: Liaoning, Jilin, Heilongjiang). The community data set of CLHLS 2018 cross sectional survey included the community information on socioeconomic, medical and older adults services, air pollution and other environmental pollution in more than 860 counties, county-level cities or districts in the country's 23 provinces, municipalities and autonomous regions, and was matched with the individual data of the Chinese Geriatric Health Survey. All the datesets can be found at https://opendata.pku.edu.cn.

**Figure 1:**
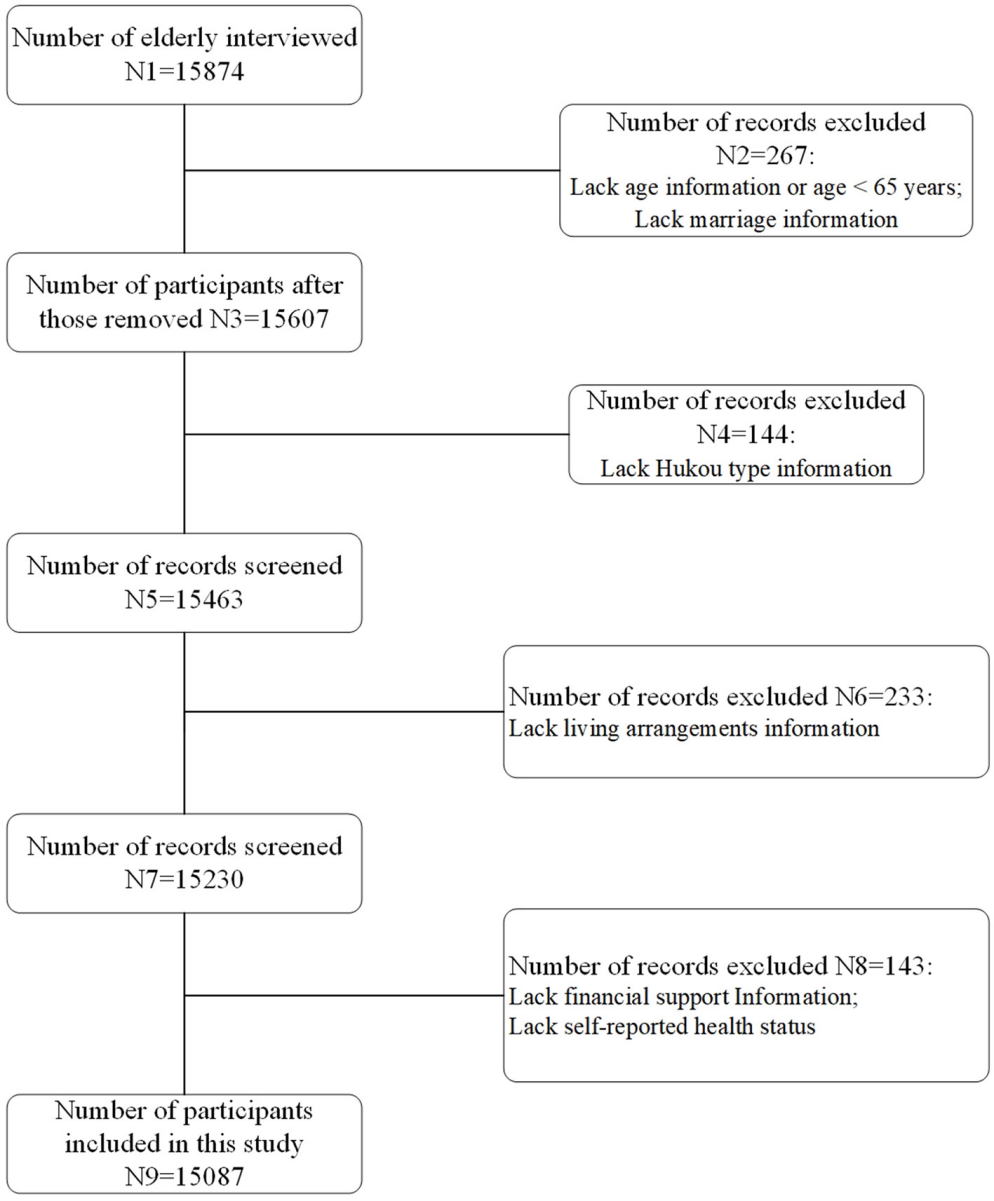
Flowchart of data selecting.

Data were obtained from the 2018 wave of cross-sectional dataset of Chinese Longitudinal Healthy Longevity Survey, which included interviews with 15,874 older adults (≥65 years). After excluding participants younger than 65 years and those with missing sociodemographic information, ultimately, 10,587 older adults individuals were selected as the research subjects for analysis (see Table 1 for detailed information, the flow chart was shown in Figure 1). Multidimensional differences were examined in older adults care services in China, by focusing on living arrangement, hukou types, economic security, service demand and accessibility under the context of population aging.

**Table 1:** Baseline characteristics by living arrangement (different living arrangement) and hukou type.

Characteristics	Total sample (*N* = 15,087)	Hukou			Living arrangement			
		Urban	Rural	*P*	With family member	Alone	In an institution	*P*
		4,203 (72.86)	10,884 (72.14)		12,131 (80.41)	2,411 (15.98)	545 (3.61)	
Sociodemographic								
Age (years)	85 (76, 95)	85 (76, 95)	85 (76, 95)	0.021	85 (75, 96)	85 (79, 92)	85 (83, 97)	< 0.001
Gender (male)	6,572	1,988	4,584	< 0.001	5,459	895	218	< 0.001
Gender (female)	8,515	2,215	6,300		6,672	1,516	327	
Marital status (currently married and living with spouse)	5,906	1,810	4,096	< 0.001	5,722	127	57	< 0.001
Marital status (separated/divorced/widowed/never married)	9,181	2,393	6,788		6,409	2,284	488	
Hukou (urban)	4,203	—	—	< 0.001	3,318	518	367	< 0.001
Hukou (rural)	10,884	—	—		8,813	1,893	178	
Mode of residence (with household members)	12,131	3,318	8,813	< 0.001	—	—	—	< 0.001
Mode of residence (alone)	2,411	518	1,893		—	—	—	
Mode of residence (in an institution)	545	367	178		—	—	—	
Living arrangement older adults preferred (living alone regardless residential distance with children)	1,770	562	1,208	< 0.001	1,293	453	24	< 0.001
Living arrangement older adults preferred (living alone (/with spouse) and children living nearby)	4,855	1,467	3,388		3,432	1,358	65	
Living arrangement older adults preferred (Co-residence with children)	7,082	1,699	5,383		6,556	412	114	
Living arrangement older adults preferred (institutions)	341	202	139		40	47	254	
Current residential area (city)	3,425	3,156	269	< 0.001	2,702	408	315	< 0.001
Current residential area (town)	4,953	854	4,099		3,983	816	154	
Current Residential area (rural)	6,709	193	6,516		5,446	1,187	76	
Residential province (Eastern region)	7,158	2,501	4,657	< 0.001	5,521	1,231	406	< 0.001
Residential province (Central region)	3,566	502	3,064		2,901	611	54	
Residential province (Western region)	3,709	799	2,910		3,145	498	66	
Residential province (Northeast region)	654	401	253		564	71	19	
Socioeconomic status								
Financial support sufficient (yes)	12,961	3,897	9,064	< 0.001	10,445	2,039	477	0.078
Financial support sufficient (no)	2,126	306	1,820		1,686	372	68	
Main source of financial support (self/spouse)	5,311	2,974	2,337	< 0.001	4,379	664	268	< 0.001
Main source of financial support [child(ren)/grandchild (ren)]	7,052	668	6,384		5,777	1,114	161	
Main source of financial support (others)	2,319	331	1,988		1,654	574	91	
Got adequate medical treatment at around age 60 (yes)	2,413	615	1,798	0.001	1,981	378	54	0.104
Got adequate medical treatment at around age 61 (no)	191	29	162		145	40	6	
Physical health status								
Health at present (very good/good)	6,519	1,903	4,616	0.009	5,273	1,041	205	< 0.001
Health at present (so so)	5,367	1,456	3,911		4,279	916	172	
Health at present (bad/very bad)	1,958	528	1,430		1,548	336	74	
Health at present (not able to answer)	1,243	316	927		1,031	118	94	
Life quality at present (very good/good)	9,683	2,895	6,788	< 0.001	8,000	1,378	305	< 0.001
Life quality at present (so so)	3,699	902	2,797		2,784	795	120	
Life quality at present (bad/very bad)	450	91	359		307	118	25	
Life quality at present (not able to answer)	1,255	315	940		1,040	120	95	
Who take care of you when you are sick (spouse)	4,086	1,269	2,817	< 0.001	3,953	106	27	< 0.001
Who take care of you when you are sick (children)	7,235	1,870	5,365		5,599	1,504	132	
Who take care of you when you are sick (others)	3,519	1,008	2,511		2,390	752	377	

#### Statistical analysis

Categorical variables were presented as numbers (percentages), and continuous variables were presented as Means (*SD*) or described as Median (*Quartiles*). Differences in the distribution of categorical characteristics among older adults with different living arrangement and hukou types were first examined by χ^2^ test. For continuous variables, the *F* test or *Kruskall–Wallis* test was used for comparison between different groups. Multiple logistic regression analysis was employed to identify the factors influencing the living arrangement of older adults, and calculate the corresponding odds ratios (*ORs*) and 95% confidence intervals (*CIs*). Then logistic regression models were performed to assess the association of hukou types between demographic characteristics, financial support and self-reported health status, etc. Differences in the distribution of availability of social security and availability of social services between older adults with different hukou types were also examined by χ^2^ test.

SPSS software version 25.0 was used for statistical analysis. *P* < 0.05 (two sides) was considered as statistically significant.

## Results

### Basic information

Among the 15,087 participants, 43.56% (*n* = 6,572) were male, and median age was 85 years (76, 95). Detailed demographic characteristics of the older adults are presented in Table 1. At the same time, 46.45% (*n* = 6,970) indicated that their main source of financial support came form their child(ren), 1.69% (*n* = 254) from grandchild(ren), 25.30% (*n* = 3,796) from their retirement wages, and 8.03% (1,205) came form work by themselves. When they were sick, their spouse (27.53%, *n* = 4,086), sons (36.62%, *n* = 5,434) and daughters (12.14%, *n* = 1,801) mainly took care of them. Proportions of participants from different regions (Eastern region, Central region, Western region and Northeast region) were 47.44, 23.64, 24.58 and 4.33% respectively.

### Living arrangement

The majority of participants' hukou type were registered rural (72.14%, *n* = 10,884). More than 80% of the respondents stated their living arrangement were living with their families (80.41%, *n* = 12,131), 15.98% living alone, and 3.61% living in an institution.

The living arrangement older adults preferred were co-residence with children (50.41%, *n* = 7,082) and living alone (/with spouse) with children living nearby (34.56%, *n* = 4,855). Very few older adults people (545, 3.61%) chose to live in an institution, among them, 42.56% (*n* = 246) indicated that the costs were paid by their sons and spouse. The main reasons that choosing living alone included no child or child is unavailable for care-giving (23.88%, *n* = 550) and don't want to bother children (63.70%, *n* = 1,467). The reasons that choosing an institution included no child or child was unavailable for care-giving (49.16%, *n* = 350) and don't want to bother children (32.58%, *n* = 232).

There was a statistical difference between the living arrangement and life quality and health at present of the interviewed older adults (*P* < 0.001; *P* < 0.001). We found that the older adults living with their household member(s) were more likely to self-report their life quality as very good and good (65.84%) than those living alone (57.00%) or living in an institution (55.55%). While the older adults living with their household member(s) were less likely to self-report their life quality as bad and very bad (2.54%) than those living alone (5.07%) or living in an institution (4.76%) as shown in Table 1.

Multiple logistic regression was used to detect the relationship between living arrangement and characteristics of the interviewed older adults, results were presented in Table 2. Urban older adults were significantly more likely to be in an institution (8.73 vs. 1.64%, *OR* = 4.782, 95% *CI* = 3.795–6.025, *P* < 0.001), less likely to be alone (13.32 vs. 17.39%, *OR* = 0.694, 95% *CI* = 0.603–0.799, *P* < 0.001), compared with rural older adults. As age increased, the older adults were less likely to be alone or in in an institution (*OR* = 0.950, 95% *CI* = 0.945–0.954, *P* < 0.001; *OR* = 0.984, 95% *CI* = 0.974–0.994, *P* = 0.001). Male older adults were more likely to be alone or in an institution than female (*OR* = 1.190, 95% *CI* = 1.072–1.322, *P* = 0.001; *OR* = 1.332, 95% *CI* = 1.095–1.622, *P* = 0.004).

**Table 2:** Multiple logistic regression on the relationship between living arrangement and characteristics of the interviewed older adults.

Living arrangement	Independent variables	β	Se	Wald	*P*	OR	95%CI	
Alone	Intercept	2.974	0.306	94.416	< 0.001			
	Age (years)	−0.052	0.003	393.021	< 0.001	0.950	0.945	0.954
	Sex = male (reference: female)	0.174	0.053	10.609	0.001	1.190	1.072	1.322
	Hukou type of the older adults being visited = urban (reference: rural)	−0.365	0.072	25.689	< 0.001	0.694	0.603	0.799
	Marriage = currently married and living with spouse (reference: separated/divorced/never married)	−3.693	0.103	1,284.424	< 0.001	0.025	0.020	0.030
	Main source of financial support = self/spouse (reference: others)	−0.391	0.081	23.041	< 0.001	0.677	0.577	0.794
	Main source of financial support = children/grandchildren (reference: others)	−0.733	0.065	126.066	< 0.001	0.480	0.423	0.546
	Health at present = very good/good (reference: not able to answer)	0.621	0.110	31.939	< 0.001	1.861	1.500	2.308
	Health at present = so (reference: not able to answer)	0.748	0.111	45.233	< 0.001	2.113	1.699	2.628
	Health at present = bad/very bad (reference: not able to answer)	0.709	0.123	33.103	< 0.001	2.033	1.596	2.588
	Residential provinces = Eastern region (reference: Northeast region)	0.751	0.143	27.674	< 0.001	2.118	1.601	2.802
	Residential provinces = Central region (reference: Northeast region)	0.557	0.148	14.090	< 0.001	1.745	1.305	2.334
	Residential provinces = Western region (reference: Northeast region)	0.155	0.149	1.076	0.300	1.167	0.872	1.563
In an institution	Intercept	−1.764	0.577	9.345	0.002			
	Age (years)	−0.017	0.005	10.380	0.001	0.984	0.974	0.994
	Sex = male (reference: female)	0.287	0.100	8.185	0.004	1.332	1.095	1.622
	Hukou type of the older adults being visited = urban (reference: rural)	1.565	0.118	176.031	< 0.001	4.782	3.795	6.025
	Marriage = currently married and living with spouse (reference: separated/divorced/never married)	−2.536	0.165	236.245	< 0.001	0.079	0.057	0.109
	Main source of financial support = self/spouse (reference: others)	−0.383	0.147	6.827	0.009	0.682	0.511	0.909
	Main source of financial support = children/grandchildren (reference: others)	−0.716	0.139	26.447	< 0.001	0.489	0.372	0.642
	Health at present = very good/good (reference: not able to answer)	−0.643	0.146	19.486	< 0.001	0.526	0.395	0.699
	Health at present = so (reference: not able to answer)	−0.467	0.151	9.544	0.002	0.627	0.466	0.843
	Health at present = bad/very bad (reference: not able to answer)	−0.381	0.179	4.525	0.033	0.683	0.481	0.970
	Residential provinces = Eastern region (reference: Northeast region)	1.293	0.245	27.779	< 0.001	3.643	2.252	5.891
	Residential provinces = Central region (reference: Northeast region)	0.290	0.281	1.065	0.302	1.336	0.771	2.316
	Residential provinces = Western region (reference: Northeast region)	0.114	0.274	0.175	0.676	1.121	0.655	1.918

The reference of residence mode was living with house members. There was 405 missing data about main source of financial support.

**Figure 2:**
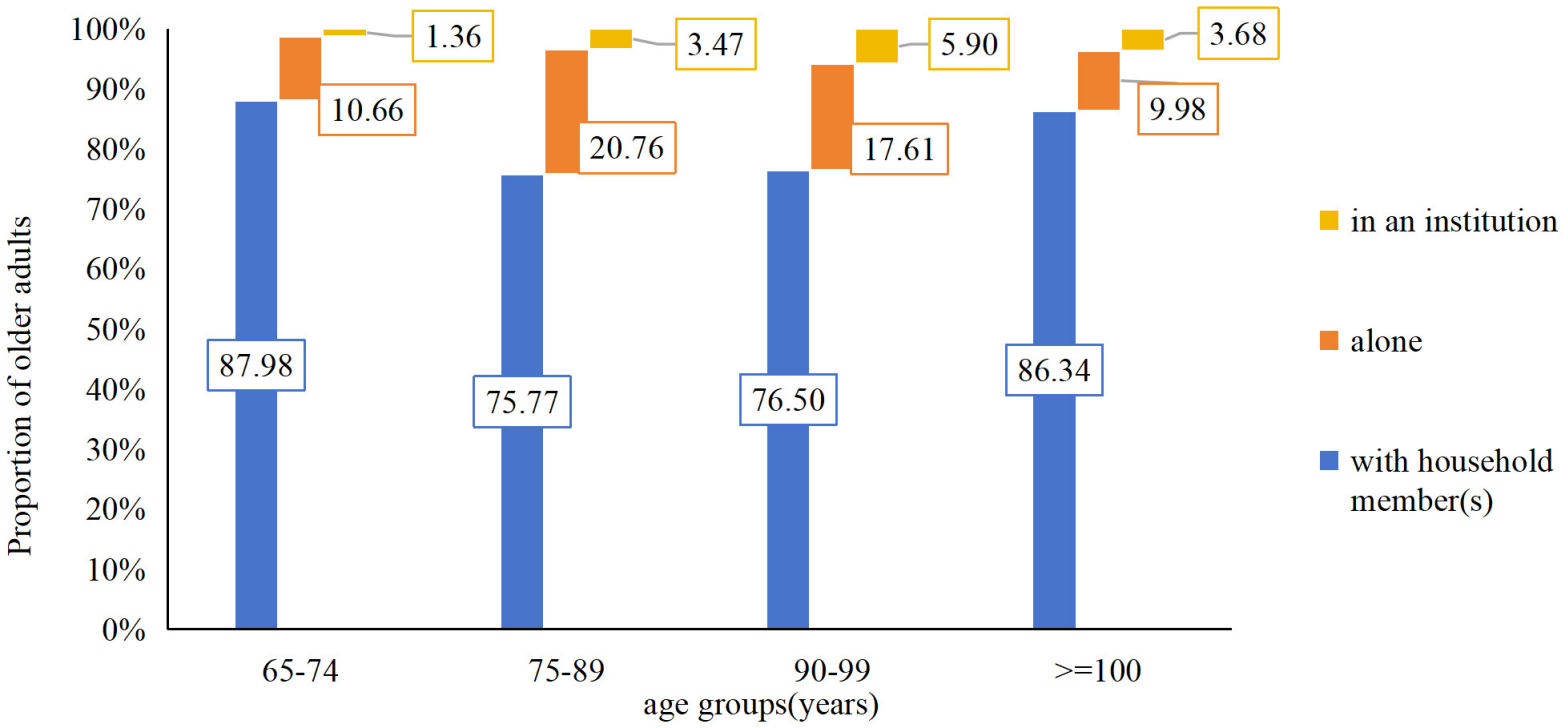
Living arrangement among different age group.

As they were getting older, the interviewed older adults were more likely to live with their household member(s) than alone or in an institution. After stratifying by age, it was found that living arrangement varied among different age groups (χ^2^3 ^=^ 56.025, *P* < 0.001). Those aged 65–74 years and above 100 years exhibited higher co-residence rates (87.88 and 86.06%, respectively) compared to those aged 75–89 years and 90–99 years (75.70 and 76.50%, respectively) as shown in Figure 2.

### Urban-rural disparities in economic status and institutional support

The proportion of urban older adults who rated their economic status as “very rich/rich” was significantly higher than those rural counterparts (29.65 vs. 15.31%, *P* < 0.001). In contrast, the proportion of rural older adults who rated themselves as “poor/very poor” was markedly higher than urban older adults (13.01 vs. 4.99%, *P* < 0.001). A higher percentage of urban older adults participated in public old-age insurance compared to rural older adults (41.09 vs. 34.28%, *P* < 0.001). Meanwhile, a greater proportion of rural older adults remained engaged in paid work after retirement than their urban peers (21.76 vs. 14.69%, *P* < 0.001). Coverage of basic national medical insurance was relatively high in both groups, reaching 63.76% among urban older adults and 82.70% among rural older adults. In contrast, commercial medical insurance coverage was extremely low in both urban and rural areas (below 1%), with no statistically significant difference between the two groups (*P* = 0.412). More details referred in Table 3.

**Table 3:** Differences between hukou type and insurance characteristics of the interviewed older adults.

Characteristics	Hukou type				Total			*P*
	Urban		Rural		*N*	Percent (%)		
	*N*	Percent (%)	*N*	Percent (%)				
Self-rated economic status compared with other local people (*N* = 14,982)	Very rich/rich	1,237	29.65	1,655	15.31	2,892	19.30	< 0.001
	So	2,727	65.36	7,749	71.68	10,476	69.92	
	Poor/very poor	208	4.99	1,406	13.01	1,614	10.77	
Public old age insurance? (*N* = 12,156)	Yes	786	41.09	3,511	34.28	4297	35.35	< 0.001
	No	1,127	58.91	6,732	65.72	7859	64.65	
Still engaged in paid jobs after retirement? (*N* = 3,783)	Yes	437	14.69	176	21.76	613	16.20	< 0.001
	No	2,537	85.31	633	78.24	3,170	83.80	
Retirement pension at present? (*N* = 13,913)	No	1,221	30.28	9,134	92.44	10,355	74.43	< 0.001
	Yes	2,811	69.72	747	7.56	3,558	25.57	
Public free medical services at present? (*N* = 13,682)	No	3,506	90.43	9,676	98.68	13,182	96.35	< 0.001
	Yes	371	9.57	129	1.32	500	3.65	
Urban employee/resident medical insurance at present? (*N* = 13,871)	No	1,441	36.24	8,986	90.81	10,427	75.17	< 0.001
	Yes	2,535	63.76	909	9.19	3,444	24.83	
New rural cooperative medical insurance at present? (*N* = 14,302)	No	3,427	87.96	1,800	17.30	5,227	36.55	< 0.001
	Yes	469	12.04	8,606	82.70	9,075	63.45	
Commercial medical insurance at present? (*N* = 13,660)	No	3,836	99.17	9,724	99.31	13,560	99.27	0.412
	Yes	32	0.83	68	0.69	100	0.73	

There were different numbers of participants who has valid information about different questions, so their “Ns” were different among different statistical tests on these questions.

Compared with urban older adults, those in rural areas were more likely to claim their main source of financial support was children/grandchild(ren) (*OR* = 1.790, 95% *CI* = 1.551–2.066, *P* < 0.001), and less likely to rely on themselves or their spouses (*OR* = 0.083, 95% *CI* = 0.072–0.095, *P* < 0.001). More details referred in Table 4.

**Table 4:** Binary logistic regression on the relationship between hukou types and main source of financial support.

Model	Main source of financial support	*P*	OR	95% CI	
1	Others	< 0.001	1.000		
	Retirement wages/work by self/spouse	< 0.001	0.131	0.115	0.149
	Child(ren)/grandchild(ren)	< 0.001	1.591	1.382	1.832
2	Others	< 0.001	1.000		
	Retirement wages/work by self/spouse	< 0.001	0.083	0.072	0.095
	Child(ren)/grandchild(ren)	< 0.001	1.790	1.551	2.066

The reference of hukou type was registered urban residents. There was 405 missing data about main source of financial support.

Model 1: unadjusted.

Model 2: adjusted by age, gender, marriage and residential provinces.

### Urban-rural disparities in availability of social/community services

There were differences in the accessibility of social/community services between older adults people in urban and rural areas. Except for home visit services, other community services were more accessible to urban older adults than rural older adults. While other community services were more required for rural older adults than urban older adults except for home visit services and healthcare education services. Only 10.31% (1,511) of the participants claimed that personal care services were available in their community, while 64.71% (*n* = 9,503) of them expected their community to provide personal care services. As shown in Table 4, access to different social services like home visit services, psychological consulting services, and daily shopping services were 34.22% (*n* = 5,041), 14.53% (*n* = 2,132), and 11.26% (*n* = 1,648), respectively. However, the demands for these services were 81.78% (*n* = 12,064), 67.93% (*n* = 9,973), and 60.93% (*n* = 8,927), respectively. The same problem existed in the supply and demand of other community services. These results suggested that there is a gap between the actual provision of and the demand for community services for the participants. More detailed results were shown in Table 5.

**Table 5:** Differences between hukou type of the older adults being visited and the availability of social/community services.

Kind of social/community services	Accessibility?	Hukou type				Total	*P*	Expectation?	Hukou type				Total	*P*
		Urban		Rural					Urban		Rural			
		*N*	Percent (%)	*N*	Percent (%)				*N*	Percent (%)	*N*	Percent (%)		
Personal care?	Yes	691	16.83	820	7.78	1,511	< 0.001	Yes	2,677	65.15	6,826	64.54	9,503	0.489
	No	3,415	83.17	9,725	92.22	1,3140		No	1,432	34.85	3,750	35.46	5,182	
	Total	4,106	100.00	10,545	100.00	1,4651		Total	4,109	100.00	10,576	100.00	1,4685	
Home visit?	Yes	1,187	28.90	3,854	36.28	5,041	< 0.001	Yes	3,152	76.28	8,912	83.92	12,064	< 0.001
	No	2,920	71.10	6,769	63.72	9,689		No	980	23.72	1,708	16.08	2,688	
	Total	4,107	100.00	10,623	100.00	14,730		Total	4,132	100.00	10,620	100.00	14,752	
Psychological consulting?	Yes	919	22.37	1,213	11.48	2,132	< 0.001	Yes	2,816	68.38	7,157	67.76	9,973	0.464
	No	3,190	77.63	9,353	88.52	12,543		No	1,302	31.62	3,406	32.24	4,708	
	Total	4,109	100.00	10,566	100.00	14,675		Total	4,118	100.00	10,563	100.00	14,681	
Daily shopping service?	Yes	598	14.58	10,50	9.96	1,648	< 0.001	Yes	2,546	61.95	6,381	60.53	8,927	0.114
	No	3504	85.42	9,488	90.04	12,992		No	1,564	38.05	4,161	39.47	5,725	
	Total	4102	100.00	10,538	100.00	14,640		Total	4,110	100.00	10,542	100.00	14,652	
Social and recreation?	Yes	1480	35.90	1,727	16.36	3,207	< 0.001	Yes	2,775	67.67	7,071	66.96	6	0.414
	No	2642	64.10	8,831	83.64	11,473		No	1,326	32.33	3,489	33.04	4,815	
	Total	4122	100.00	10,558	100.00	14,680		Total	4,101	100.00	10,560	100.00	14,661	
Legal aid?	Yes	1318	31.95	1,724	16.33	3,042	< 0.001	Yes	2,714	66.15	6,884	65.34	9,598	0.359
	No	2807	68.05	8,834	83.69	11,641		No	1,389	33.85	3,651	34.66	5,040	
	Total	4125	100.00	10,555	100.00	14,683		Total	4,103	100.00	10,535	100.00	14,638	
Healthcare education?	Yes	1894	45.80	4,272	40.15	6,166	< 0.001	Yes	3,015	73.36	8,133	76.91	11,148	< 0.001
	No	2241	54.20	6,368	59.85	8,609		No	1,095	26.64	2,442	23.09	3,537	
	Total	4135	100.00	10,640	100.00	14,775		Total	4,110	100.00	10,575	100.00	1,4685	
Neighborhood-relation?	Yes	1638	39.59	2,994	28.34	4,632	< 0.001	Yes	2,782	67.84	7,218	68.57	10,000	0.390
	No	2499	60.41	7,572	71.66	10,071		No	1,319	32.16	3,308	31.43	4,627	
	Total	4137	100.00	10,566	100.00	14,703		Total	4,101	100.00	10,526	100.00	14,627	
**Any other social?**	Yes	447	11.62	737	7.73	1,184	< 0.001	Yes	1,548	40.16	4,266	44.87	5,814	< 0.001
	No	3401	88.38	8,797	92.27	12,198		No	2,307	59.84	5,241	55.13	7,548	
	Total	3848	100.00	9,534	100.00	13,382		Total	3,855	100.00	9,507	100.00	13,362	

There were different numbers of participants who has valid information about different questions, so their “Ns” were different among different statistical tests on these questions.

The binary logistic regression results indicated that hukou type exerted a statistically significant effect on older adults care service demands (see Table 6). Compared with those rural older adults, urban older adults exhibited significantly higher likelihoods of demanding most types of services, including personal care, psychological consulting, daily shopping assistance, social and recreational activities, legal aid, healthcare education, and neighborhood-related services, with odds ratios generally exceeding 1. In contrast, the demand for home visit services was significantly lower among urban older adults (*OR* < 1), suggesting a relative reliance on such basic services in rural areas. These patterns remain robust across both models, even after controlling for additional variables, although the magnitude of the effects was moderately attenuated.

**Table 6:** Binary logistic regression on the relationship between hukou types and older adults care services.

Model	Older adults care services	Hukou type	*P*	OR	95% CI	
1	Personal care	Urban	< 0.001	2.400	2.153	2.675
		Rural		1.000		
	Home visit	Urban	< 0.001	0.714	0.660	0.772
		Rural		1.000		
	Psychological consulting	Urban	< 0.001	2.221	2.021	2.442
		Rural		1.000		
	Daily shopping service	Urban	< 0.001	1.542	1.385	1.717
		Rural		1.000		
	Social and recreation	Urban	< 0.001	2.864	2.639	3.109
		Rural		1.000		
	Legal aid	Urban	< 0.001	2.406	2.214	2.615
		Rural		1.000		
	Healthcare education	Urban	< 0.001	1.260	1.172	1.354
		Rural		1.000		
	Neighborhood-relation	Urban	< 0.001	1.658	1.537	1.787
		Rural		1.000		
2	Personal care	Urban	< 0.001	1.905	1.668	2.176
		Rural		1.000		
	Home visit	Urban	< 0.001	0.723	0.660	0.792
		Rural		1.000		
	Psychological consulting	Urban	< 0.001	2.256	2.048	2.486
		Rural		1.000		
	Daily shopping service	Urban	< 0.001	1.518	1.359	1.695
		Rural		1.000		
	Social and recreation	Urban	< 0.001	2.351	2.130	2.594
		Rural		1.000		
	Legal aid	Urban	< 0.001	2.104	1.906	2.322
		Rural		1.000		
	Healthcare education	Urban	0.006	1.129	1.036	1.232
		Rural		1.000		
	Neighborhood-relation	Urban	< 0.001	1.566	1.431	1.714
		Rural		1.000		

Missing data of persona care, home visit, psychological consulting, daily shopping service, social and recreation, legal aid, healthcare education and neighborhood-relation were 436, 357, 412, 447, 407, 404, 312 and 384 respectively.

Model 1: unadjusted.

Model 2: adjusted by age, gender, marriage, financial status and living arrangement.

## Discussion

### Key findings

Results revealed that the majority of older adults resided with family members, while only a small proportion (3.61%) chose to live in institutions. This pattern aligned with the traditional Chinese concept of filial piety, where children were expected to take care for their older adults parents (21). However, residential arrangements also exhibited age-related structural differences (see Figure 2). This U-shaped distribution might be related to the fact that younger older adults people still had the ability to work at home, and centenarians had an increasing demand for care and companionship, further strengthened the dominant position of family care. In addition, economic factors were also important variables that constrained institutional older adults care choices. The average monthly pension for urban and rural residents in China in 2024 was only about 246 CNY, which was difficult to meet basic living needs, let alone to pay for institutional pension expenses.

### Inter-generational financial support and family burden

Consistent with prior research, it was observed that living arrangement exerted a significant influence on the quality of life and health of older adults (22). This suggested that co-residence was associated with better perceived life quality. Existing research corroborated that home-based care played a more fundamental role than institutional care within China's eldercare policy framework (23). Furthermore, studies indicated that health disparities among Chinese older adults were primarily driven by inequalities in income, medical expenses, and living arrangement (24). The preferred living arrangements of the older adults were: living with their children (50.41%) and living alone or with their spouse near their children (34.56%), further confirming the practical significance of family support. Studies had also shown that frequent visits by children were associated with a reduced risk of cognitive impairment in the older adults, reflecting the comprehensive benefits of family companionship on physical and mental health (25–28).

Data showed that 46.45% of older adults people mainly relied on their children for financial support. In addition, nearly half of the older adults care expenses in institutions were borne by their children or spouses (44.73%), exacerbating the economic burden on families. It was consistent with existing research findings that the economic ability of children had a decisive impact on the accessibility of inter-generational support (29). It further emphasized that economic factors were the real obstacles that constrained the choice of elder's living arrangement (30–32).

### Urban-rural disparities in older adults care and social security

**Figure 3:**
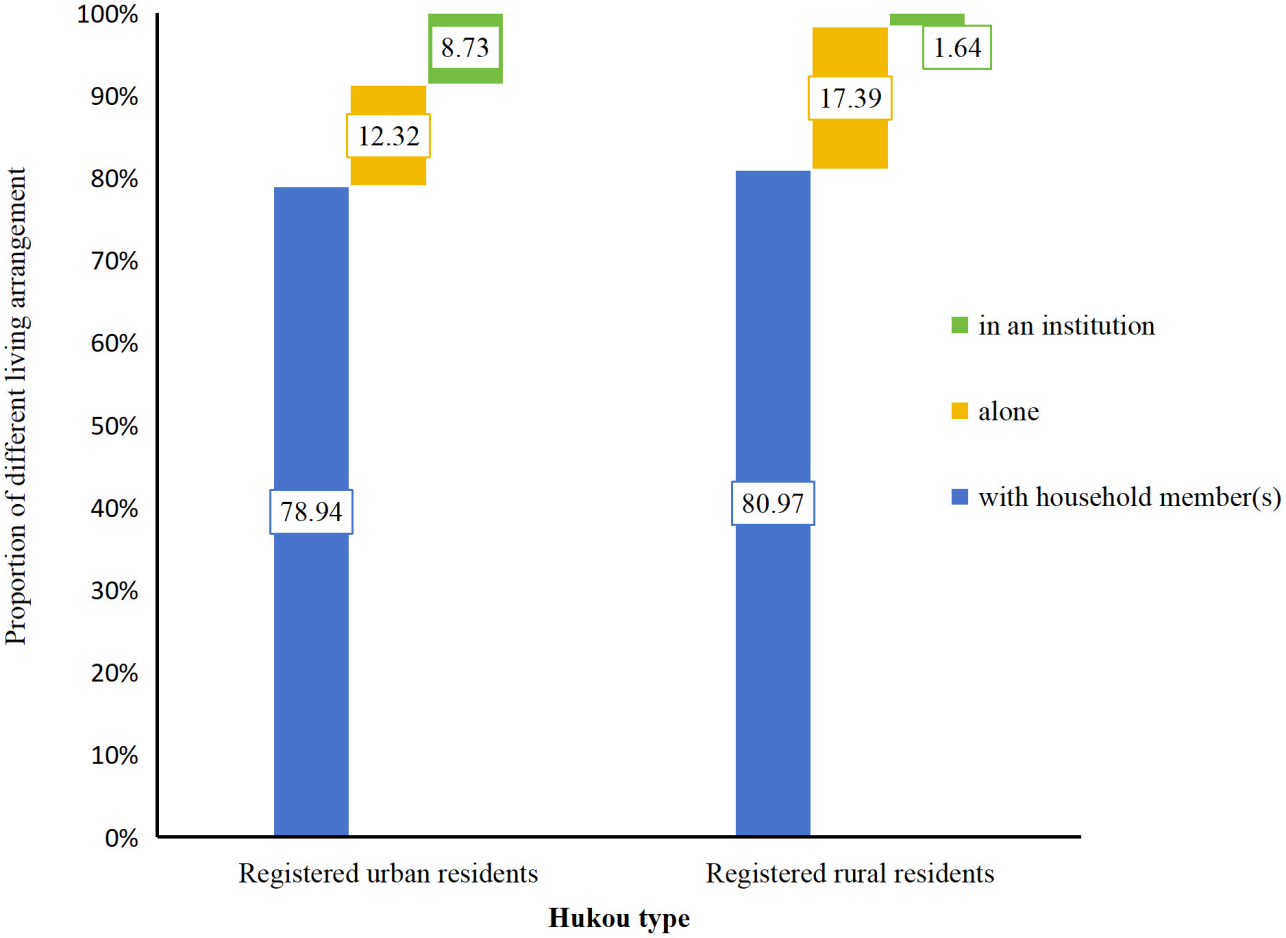
Living arrangement between older adults with different Hukou types.

Previous research confirmed that many older adults faced concurrent social, emotional, financial, and medical challenges (33). While emotional support came mainly from spouses and children, financial support relied predominantly on children. Consequently, those in better financial situations (mainly supported by children) showed a greater propensity to utilize institutional care (34, 35). Results in this study showed urban older adults had better economic conditions than rural older adults, their financial support relied mainly on themselves, while rural older adults mainly relied on their children. It was consistent with social support theory, rural older adults will seek alternative support sources when facing insufficient financial assistance. Results also showed the proportion of urban older adults living in institutions was higher than rural older adults, while less of urban older adults engaged in paid jobs than rural older adults. Figure 3 showed a markedly higher institutionalization rate among urban residents compared to rural residents (9.48 vs. 1.15%, *P* < 0.001), attributable to the substantial urban-rural gap in socioeconomic development (36).

### Mismatch between demand and supply of older adults care services

**Figure 4:**
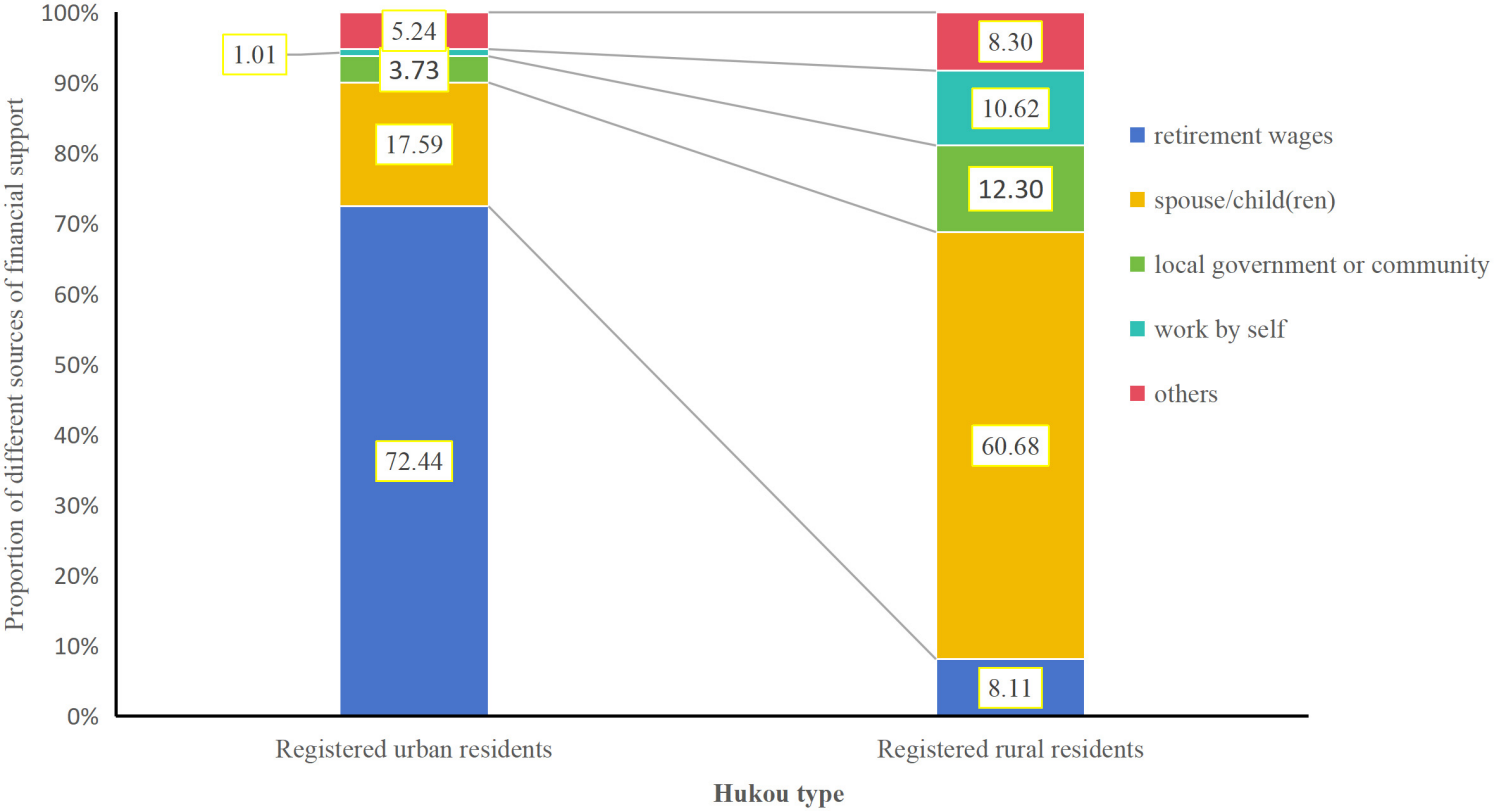
Main source of financial support between older adults with different Hukou types.

Given the often constrained economic resources of older adults, accessing support within their home communities proved more feasible and satisfactory than institutionalization (37). However, a critical finding was the significant mismatch between the demand for community old-age-services and their actual provision (see Tables 4, 5). According to previous studies (6, 23), this mismatch manifested in three key areas: (I) Supply-demand imbalance: a structural contradiction existed between strong actual demand and inadequate effective supply of old-age-services. The layout and planning of older adults care service facilities had not fully considered the living habits and actual needs of the older adults, resulting in a coexistence of idle and insufficient supply of older adults care facilities. (II) Content disconnect: the older adults care services offered often failed to align with the specific needs expressed by older adults. The inaccurate grasp of the real needs of the older adults by service providers had led to a focus on basic life care in service provision, while the supply of diversified needs such as medical care, spiritual comfort, and cultural entertainment was insufficient. (III) Urban-rural disparity: the development of old-age-services remained highly uneven across urban and rural regions. Consistent with it, our study further revealed urban-rural differences on community service accessibility: urban older adults enjoyed greater access to available community services. And urban older adults expressed higher demand for services meeting advanced needs (e.g., social activities), while rural older adults prioritized basic needs (e.g., healthcare, home visits). Therefore, we suggested that the government taking measures to narrow this gap, while providing differentiated older adults care services based on the characteristics of different regions and the different needs of the older adults to meet their actual needs. As rural older adults primarily relied on familial supports while urban older adults had sufficient income by themselves, (the difference of financial source between rural and urban older adults was shown in Table 3 and Figure 4), it should also be valued about how to ensure the financial support of the older adults and alleviated the financial pressure on their caregivers.

**Figure 5:**
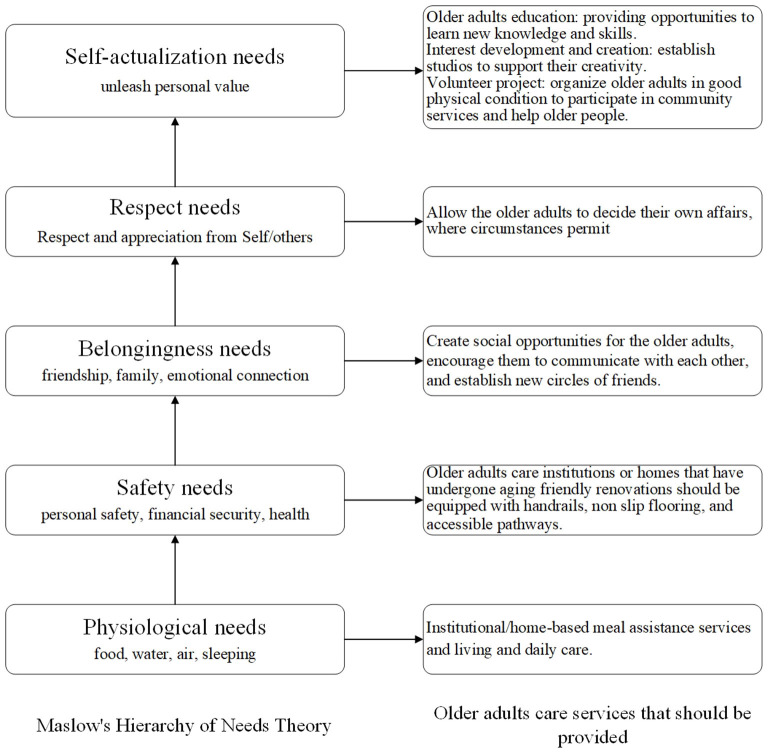
Older adults care services that should be provided based on Maslow's Hierarchy of Needs Theory.

At present, in the classification of China's older adults care service industry, older adults care services are divided into home-based older adults care services, community older adults care services, and institutional older adults care services (38). Yet, these industries were not fully developed. Rural areas placed greater emphasis on the older adults care concept of staying in the villages and hometowns. Community older adults care was the main focus of urban older adults people, but the service supply was scattered and there was a shortage of professional nursing staff. According to activity theory of aging (39), successful older adults life stemmed from active participation in society and life. Ecological theory also proposed that individual development was nested within a series of interrelated environmental systems (including micro, meso, outer, macro, and temporal systems). Therefore, policy makers should consider the social interaction needs of the older adults, the need for family companionship, and the need for self-worth realization when formulating content related to older adults care services. For example, implementing older adults friendly and barrier free renovation of public facilities for the older adults, providing psychological health services for the older adults, and encouraging the older adults to participate in older adults volunteer activities. Older adults care services designed in alignment with Maslow's hierarchy of needs according to the findings above were illustrated in Figure 5, demonstrating how various service levels correspond to physiological, safety, social, esteem, and self-actualization needs.

### Insurance coverage gaps and public awareness issues

A concerning finding was the low coverage of private/commercial pension insurance between urban and rural participants (0.83 vs. 0.69%). And there was no statistically significant difference in the prevalence of commercial medical insurance between urban and rural areas (*P* = 0.412), indicating that it has not yet been popularized among the older adults population. Although urban seniors were more likely to have public pension insurance than rural seniors (41.09 vs. 34.28%, *p* < 0.001), the primary reasons for non-participation were perceived lack of necessity (22.50%), affordability (21.79%), and lacking of awareness (34.81%). Similar to challenges within the healthcare system (40), where substantial out-of-pocket expenses arisen due to variable insurance coverage and deductibles, a balanced and sustainable pension system must be adjustable, accessible, and, crucially, affordable. China's pension system currently comprised three pillars: public pensions (first pillar), occupational pensions (second pillar), and individual pensions (third pillar). Although the three-pillar framework had been established, it relied overwhelmingly on the first pillar, in stark contrast to developed nations like the United States, where the third pillar contributed over 30% (41). In China, the third pillar of the pension system remained marginal, underscoring the urgency of developing a more balanced and integrated multi-pillar framework. Such development required active engagement from both governmental and societal actors. Moreover, individuals' perceptions of pension responsibility—and their subsequent participation in pension insurance—were conditioned by factors such as residential context and information accessibility. Strengthening this relationship entailed institutional refinement, enhanced public communication, and sustained educational efforts aimed at reshaping traditional understandings of retirement security. Through these measures, the coordinated evolution of basic and commercial pension schemes could be achieved, and the construction of a comprehensive, equitable, and sustainable multi-level pension security system could be advanced.

### Policy implications

At present, urban–rural disparities in China's older adults care services were primarily reflected in the supply of older adults care service resources and the economic pressures faced by the older adults. The large-scale migration of young people from rural areas to cities had resulted in a loss of labor force and an increasing number of empty-nest older adults. Consequently, rural families were becoming smaller, more dispersed, and less capable of providing sustained care and support. Moreover, the limited coverage of rural older adults care institutions and services, combined with the reluctance or difficulty of older adults to leave their hometowns, made it challenging to ensure adequate care solely through family or institutional means. In this context, the rural home-based older adults care model, which positioned the family as the core and the community as the service radius, represented a practical and context-sensitive approach.

The challenges posed by population aging were comprehensive, systemic, and long-term in nature. Addressing these challenges required coordinated efforts among the government, market, society, and families to build an inclusive, healthy, and dynamic aging-friendly society. Currently, governments should prioritize the establishment of robust pension systems and the expansion of accessible community-based older adults care services. In addition, local authorities should implement targeted measures—such as policy advocacy and subsidized premiums—to increase participation in public old-age insurance programs. Policy incentives, including tax relief and financial compensation, should also be provided to family or community caregivers engaged in older adults care services.

Furthermore, it was essential to develop needs-based service models that accurately reflect the diverse requirements of the older adults population, focusing on the integration of home- and community-based care. Particular attention should be paid to aligning services with the physical, emotional, and social needs of older adults. Finally, this study highlighted the urgent need to establish standardized frameworks and quality assurance mechanisms for older adults care services, in order to accelerate the development and delivery of diversified, high-quality care for China's aging population.

## Conclusions

Given the cross-sectional design, causal inferences cannot be established, it was still found that the primary living arrangement for older adults in China was co-residence with family members currently. At the same time, significant urban–rural differences exist, primarily reflected in economic status, insurance systems, and the availability of older adults care services. In particular, the gap in older adults care services between urban and rural areas was evident in the following aspects: the limited coverage of rural care institutions and community-based services, and the greater economic pressure faced by rural older adults, who rely more heavily on informal labor income and have insufficient access to institutional support.

Population aging is not merely a “care” issue—it is a systemic, comprehensive, and long-term strategic challenge that affects sustainable economic development, social stability and harmony, inter-generational equity, and national competitiveness. Addressing this challenge requires coordinated efforts among the government, the market, society, and families.

### Generalisability

The generalisability of the findings should be considered with caution. The results are primarily applicable to older adults in China, particularly those represented in the dataset. Cultural factors, such as filial piety, play an important role in shaping living arrangements and may limit the comparability of these findings in different sociocultural contexts. The research findings have certain generalizability in Asian countries with similar cultural and filial piety backgrounds However, variations in welfare systems and older adults care infrastructures across countries may further constrain the external validity of the results. Therefore, caution is warranted when extending these findings to other populations, age cohorts, or institutional settings.

### Limitations

Despite the contributions of this study, several limitations should be acknowledged.

First, the cross-sectional design precluded causal inferences regarding the relationship between living arrangement, hukou type and the economic and insurance characteristics of older adults. Longitudinal studies are needed to examine how these disparities evolve over time.

Second, the data were based on self-reported measurements, which might be subject to recall bias or subjective interpretation, particularly for variables such as self-rated economic status. The exclusion of incomplete cases may have resulted in potential selection bias. In addition, the data were derived from the 2018 cross-sectional survey of CLHLS, which may not fully reflect the current situation, potentially leading to deviations between the research findings and contemporary realities.

Third, although this study used nationally representative data, the findings may not be fully generalizable to all regions in China, given the substantial heterogeneity in economic development and policy implementation across provinces. the applicability of findings to older adults in other regions of China not fully captured in the dataset. Multi-regional studies or subgroup analyses by regions are recommended to capture this diversity.

Finally, this study focused primarily on economic and institutional dimensions of older adults care, with limited attention to psychosocial factors such as social support, mental health, or quality of life. Future research should adopt a more holistic framework to better understand the multidimensional needs of the aging population.

Despite these limitations, this study provides valuable insights into the structural disparities between urban and rural older adults in China and offers empirical evidence to inform targeted policy interventions.

## Data Availability

Data we used in this article was obtained from the Chinese Longitudinal Healthy Longevity Survey (CLHLS), which was organized by the Development Research Center of Health and Aging (the National Development Research Institute) of Peking University.
